# Evaluation of Eating Quality in Japonica Rice: A Multi-Trait Analysis of Starch Properties, Protein Content and Taste Value

**DOI:** 10.3390/foods15101689

**Published:** 2026-05-12

**Authors:** Yuqianqian Li, Meng Li, Jiayuan Chang, Kaiwen Gu, Jing Yu, Xiaoming Zhang, Jinsong Bao

**Affiliations:** 1 Institute of Nuclear Agricultural Sciences, Key Laboratory of Nuclear Agricultural Science of Ministry of Agriculture and Rural Affairs, College of Agriculture and Biotechnology, Zhejiang University, Zijingang Campus, Hangzhou 310058, China; liyuqianqian@zju.edu.cn (Y.L.); limeng200207@163.com (M.L.); 22416002@zju.edu.cn (J.C.); 22516184@zju.edu.cn (K.G.); yjingyx@163.com (J.Y.); 2 Institute of Crop and Nuclear Technology Utilization, Zhejiang Academy of Agricultural Sciences, Hangzhou 310021, China

**Keywords:** eating quality, taste value, protein content, gelatinization temperature, physicochemical property

## Abstract

Rice eating quality is a core determinant of consumer preference and commercial value. Although it is chemically determined by the accumulation and distribution of the substances in rice seeds, the comprehensive physicochemical basis underlying this trait in japonica rice remains insufficiently clarified. To identify the key physicochemical indicators that predict and regulate japonica rice eating quality, the taste value of 59 japonica rice varieties was evaluated, and the protein content (PC), apparent amylose content (AAC), starch pasting properties, gelatinization characteristics, and textural attributes were systematically measured. The results indicated that japonica rice has an average taste value of 72.0 with a range between 54.0 and 87.8. The taste value was significantly negatively correlated with PC, onset (To), peak (Tp) and conclusion (Tc) gelatinization temperatures, but was significantly positively correlated with appearance score, mouthfeel score, hot paste viscosity (HPV), and cool paste viscosity (CPV). PCA further indicated that AAC, HPV, CPV, peak viscosity (PV), and setback value (SB) were the major contributors to the first principal component, explaining 38.5% of the total variation. Stepwise regression analysis showed that the best regression equation for predicting taste value was: Taste value = 142.526 − 5.226 PC − 0.425 To (R^2^ = 0.455; *p* < 0.001), confirming PC and To as the core parameters accounting for 45.5% of the taste value variation. Path analysis further indicated that PC and To affected japonica rice eating quality through direct and indirect pathways. These findings suggest that low PC, low gelatinization temperature, high HPV, and high CPV can serve as good physicochemical indicators for the breeding of high-eating-quality japonica rice.

## 1. Introduction

Rice is the staple food of more than half of the world’s population, providing 21% of global caloric intake, with 90% of rice production originating from Asian countries [1]. With the improvement of living standards and socioeconomic development, an increasing number of consumers are pursuing high-quality rice with good taste and high nutritional value [2]. High yield and high quality are the two core objectives of rice breeding programs, and the synergistic improvement of yield and quality is the top priority in current rice breeding research. Rice quality encompasses appearance quality, milling quality, nutritional quality, and cooking and eating quality (CEQ), among which the improvement of CEQ is of foremost importance in the rice industry [2,3].

Rice eating quality is chemically determined by the composition, distribution and accumulation of substances in rice seeds. Rice grain consists of brown rice and the hull [4], and brown rice consists of rice bran, the embryo, and the endosperm [5]. Rice bran surrounds the embryo and endosperm and accounts for about 8–10% of the mass of brown rice, but it contains more than half of the total nutrient content, like flavonoids, phenolic acids, vitamin A, iron, zinc, selenium and phytic acid, etc. [5]. The brown rice can be further milled to remove the bran layer to obtain milled rice [4]. The endosperm is rich in starch and protein and is the main edible part. Starch is the primary storage substance in the endosperm, accounting for 72% to 82% (dry basis) of brown rice [6]. Protein is the second most abundant storage substance in rice after starch [7], accounting for 8–10% of the dry weight of brown rice [8]. The embryo accounts for about 2–3% of the mass of brown rice and is enriched with a variety of physiologically active compounds, like γ-aminobutyric acid, phenolic acids, γ-oryzanol, vitamin E, zinc and selenium, etc. [5].

Rice eating quality is a comprehensive evaluation of the color, odor, taste, stickiness, hardness, palatability and cold rice texture of cooked rice [6], which directly affects consumers’ final acceptance and represents the most important commercial value of rice [9]. To date, there are three main methods for evaluating rice eating quality. The first is the sensory evaluation method, in which panelists conduct a comprehensive sensory assessment of the cooked rice’s color, odor, taste, stickiness and hardness through visual observation, olfactory perception and taste testing [10,11]. This method can accurately and directly evaluate rice eating quality but has high requirements for the professional competence of panelists and is also susceptible to subjective bias. The second is the physicochemical index method, which indirectly evaluates rice eating quality through determining apparent amylose content (AAC), viscosity and gelatinization temperature [12,13,14,15,16]. The third is the instrumental evaluation method, which uses an instrument such as the rice taste analyzer manufactured by Japan’s Satake Corporation to measures the taste value [17,18,19,20]. This method calculates the rice taste value by simulating human sensory evaluation through the determination of key physicochemical indices via near-infrared spectroscopy [19,20]. It requires cooking the rice, and it is difficult to achieve continuous detection of a large number of rice samples [19].

There have been many reports on the relationship between the physicochemical properties and eating quality of rice. AAC and starch fine structure significantly correlated with pasting parameters, swelling power and solubility, while protein content (PC) had a close relation with taste analyzer parameters [16]. Xu et al. [11] indicated that AAC and PC had contrasting effects on all the sensory attributes in japonica rice, and the overall sensory quality could be predicted by the PC and gel hardness. Qin et al. [10] used a regression equation describing the relationship between the eating quality scores and physicochemical indices, in which five factors such as amylose content, water absorption, expansion rate gel, consistency and fat content were revealed. This study only used six varieties, however. Matsue et al. [21] used 33 rice cultivars to study the relationship between overall eating quality, physicochemical properties, and rice market prices and found that taste contributed the most to overall eating quality, followed by stickiness and appearance, and aroma contributed the least. They also indicated that no significant relationship was found between protein and amylose contents and eating quality, which contradicts the common knowledge that low-amylose-content and low-protein-content rice has better eating quality [7]. Therefore, it is necessary to identify the factors that determine rice eating quality.

To fulfill this objective, a total of 59 japonica rice varieties were employed to determine the relationship between their starch physicochemical properties and eating quality, and multiple regression and path analyses were used to identify the major factors. This study aims to provide a more reliable theoretical basis for eating quality and lay a research foundation for the breeding of high-quality japonica rice varieties.

## 2. Materials and Methods

### 2.1. Materials

A total of 59 japonica rice varieties were used in this study (Table S1). All the rice varieties were grown in the same field in a completely randomized design with two replications. The rice was sown in late May 2024, transplanted in late June 2024, and harvested from late September to early October 2024 at an experimental farm of Zhejiang University Zijingang Campus, Hangzhou, China. Fertilizer and field management followed conventional rice cultivation practices.

### 2.2. Rice Flour Preparation

Harvested rice grains were air-dried under natural sunlight and stored at room temperature for approximately two months. Paddy rice was dehulled using a huller (Type THU, Satake Co., Tokyo, Japan), polished using a rice mill (Type TM05C, Satake Manufacturing, Suzhou, China), and then ground into rice flour using a cyclone mill (FOSS, Aalborg, Denmark).

### 2.3. Taste Value Test

The appearance score, mouthfeel score and taste value of cooked rice were automatically determined using a rice taste analyzer (STAIA, Satake Corp., Hiroshima-ken, Japan). A total of 12 g of milled rice was weighed into a stainless steel tank and rinsed three times with pure water. Distilled water was added according to the rice-to-water ratio specified in NY/T 3837-2021 Method for Sensory Evaluation of Cooked Rice [22]. The rice was soaked for 30 min, then steamed for 30 min in an electric rice cooker. After cooking, the rice was simmered for an additional 10 min. The stainless steel tank was taken out, and the cooked rice was gently stirred to make it fluffy without agglomeration. The rice was cooled for 20 min. A total of 8 g of cooked rice was weighed and placed into a stainless steel sample ring, pressed once with a plate in the forward and reverse directions, and the rice was placed into the taste analyzer for determination. Each sample was tested with four replications.

### 2.4. AAC and PC

AAC was determined by the iodine colorimetric method [12] with four replications per sample. The absorbance of the solution at 620 nm relative to the blank solution was measured using a spectrophotometer. A standard curve was made from five standard rice samples with known amylose content, i.e., BP025 (17.0%), BP037 (28.5%), BP595 (2.0%), BP608 (8.0%), and SN05 (22.0%) [23]. A calibration curve was constructed with a coefficient of determination (R^2^) of 0.9912 (*p* < 0.001). PC was determined with two replicates by the Kjeldahl method with the conversion factor of 6.25.

### 2.5. Pasting Properties

Starch pasting properties were determined using a Rapid Visco-Analyzer (RVA, Model 4500, Perten Instrument, Hägersten, Sweden) with two replications. Rice flour (3 g, 12% moisture basis) was mixed uniformly with 25 g ddH_2_O in an RVA sample can. The Rice method in the TCW3 (Thermocline for Windows) software was used for the analysis. Pasting parameters were recorded or calculated by the Thermocline for Windows version 3 (TCW3) software. All viscosities are measured in Rapid Visco Units (RVU).

### 2.6. Textural Properties

After RVA analysis, the aluminum can containing rice flour gel was sealed with parafilm and stored at 4 °C overnight. Textural properties were measured using a Texture Analyzer (XTC-18, Shanghai Boxin Industrial Development Co., Ltd., Shanghai, China) with a standard two-cycle compression procedure according to the method described by Gao et al. [12]. Four replications were performed for each gel. A probe with a diameter of 5 mm was used to compress the gel by 10 mm at a test speed of 1 mm/s, with four replications per sample. Hardness (HD, g) was defined as the maximum peak force recorded during the first compression cycle, which was recorded by the instrument software.

### 2.7. Gelatinization Properties

Starch thermodynamic properties were analyzed using a Differential Scanning Calorimeter (DSC, Model Q20, TA Instruments, New Castle, DE, USA) with two replications. The method described by Peng et al. [23] was used with minor modifications: 2 mg of rice flour (12% moisture content) and 6 μL of distilled water were added to an aluminum crucible, which was then hermetically sealed and stored at 4 °C overnight. The aluminum crucible was placed into the DSC and equilibrated at 30 °C for 5 min, then heated from 30 °C to 110 °C at a heating rate of 10 °C/min, with an empty crucible as the blank control. Each sample was replicated two times. The onset gelatinization temperature (To), peak gelatinization temperature (Tp), conclusion gelatinization temperature (Tc) and gelatinization enthalpy (ΔH) were calculated by the Universal Analysis 2000 software.

### 2.8. Statistical Analysis

Pearson correlation analysis, hierarchical cluster analysis and principal component analysis (PCA) were performed using Origin 2024. Multiple regression analysis and path analysis were conducted in SPSS 26.0 statistical software.

## 3. Results

### 3.1. Eating Quality

**Figure 1:**
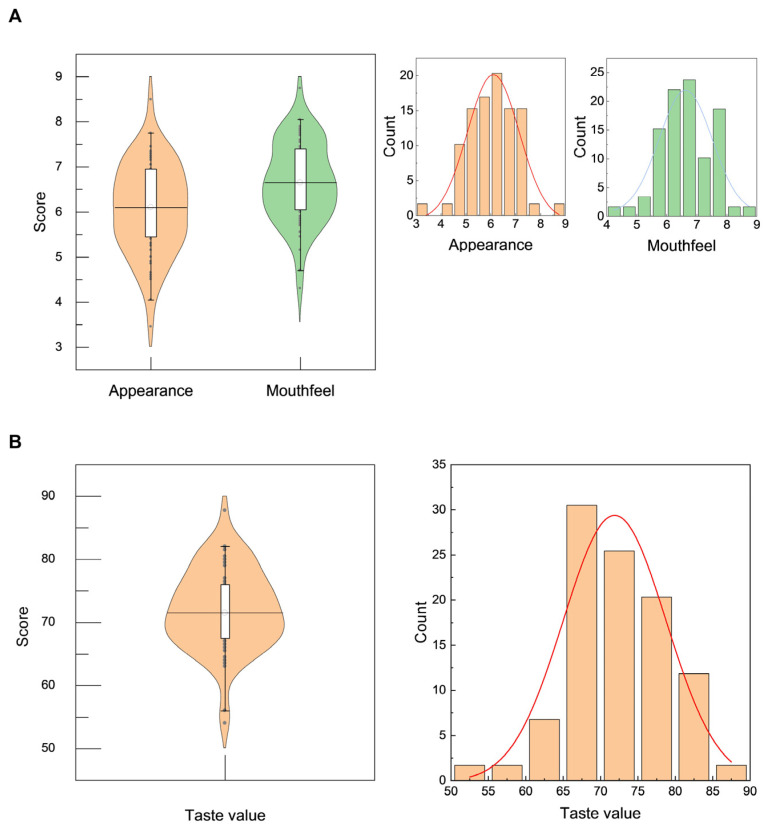
Distribution of appearance score, mouthfeel score, and taste value in different rice varieties. (**A**) Violin distribution map and histogram distribution map of appearance score and mouthfeel scores for different rice varieties (score range 0–10); (**B**) Violin distribution map and histogram distribution map of taste value for different rice varieties (score range 0–100).

The rice taste analyzer enables rapid and accurate evaluation of the eating quality of rice. The appearance score, mouthfeel score and taste value of 59 japonica rice varieties were determined using the rice taste analyzer. The results are shown in Figure 1 and Table 1. The distribution of appearance and mouthfeel scores of the tested rice varieties was relatively concentrated (Figure 1A). The histogram further confirmed that the appearance scores were mainly concentrated in the range of 4.5–8.0 points, indicating that most rice varieties exhibited good appearance traits such as grain shape and transparency. The mouthfeel scores were also concentrated in the medium-to-high range (5.5–8.0 points) with no obvious low-value outliers, suggesting that the tested rice varieties generally had good textural traits such as stickiness and elasticity. The taste values of most rice varieties were concentrated in the range of 60–85 points (Figure 1B), demonstrating that most varieties had a medium-to-high eating quality. The histogram further showed that the largest number of rice varieties had taste values in the range of 65–80 points, with very few varieties exceeding 85 points or falling below 55 points. The taste values of the tested rice varieties ranged from 54.0 to 87.8 with a mean of 72.0 (Table 1), and the variation span of more than 30 points indicated significant genetic variation in eating quality traits among different rice varieties.

### 3.2. Chemical Components

**Figure 2:**
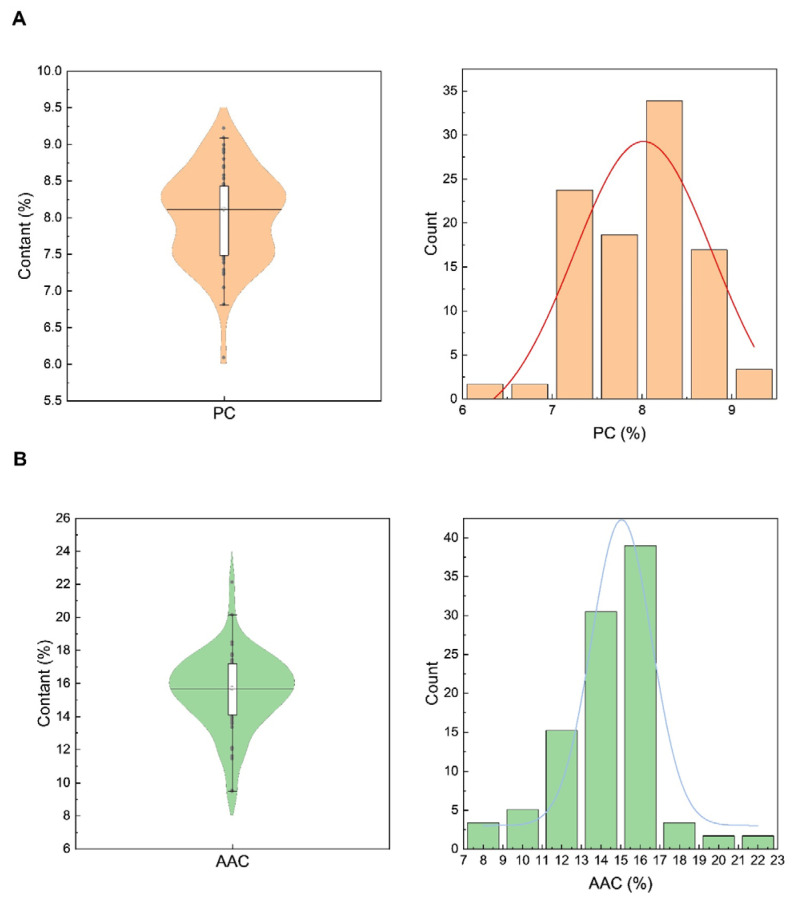
Distribution of PC and AAC in different rice varieties. (**A**) Violin distribution map and histogram distribution map of PC in different rice varieties; (**B**) Violin distribution map and histogram distribution map of AAC in different rice varieties. AAC: apparent amylose content; PC: protein content.

Figure 2A shows the distribution of total PC among 59 japonica rice varieties. The violin plot indicated two concentrated distribution ranges of total PC: 7–7.5% and 8–8.5%. The histogram further confirmed that total PC was mainly concentrated in the range of 7–8.5%, demonstrating that most rice varieties maintained a medium level of total PC. The total protein content ranged from 6.1% to 9.2% with a mean of 8.0% (Table 1). AAC was mainly concentrated in the range of 14–18% (Figure 2B), with the overall range from 9.4% to 22.1% and a mean of 15.5% (Table 1). Among the tested varieties, ultra-low-AAC varieties (6–10%) accounted for 3.4%, low-AAC varieties (10–20%) accounted for 93.2%, and medium-AAC varieties (20–25%) accounted for 3.4%, indicating that the majority of the tested rice varieties had low AAC levels.

### 3.3. Rheological Properties

**Figure 3:**
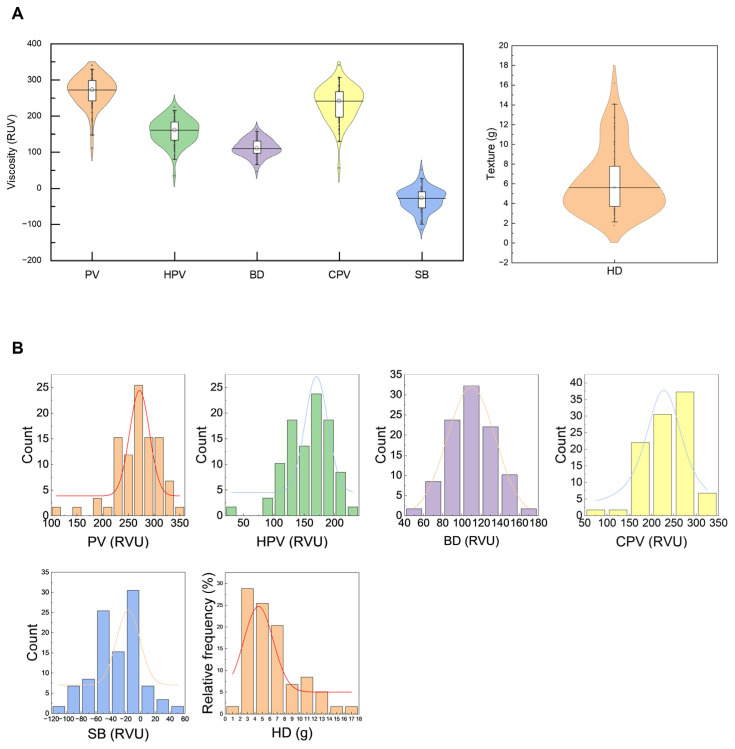
Distribution of the pasting viscosity parameters, PV, HPV, BD, CPV, SB, and HD, in different rice varieties. (**A**) Violin distribution map; (**B**) Histogram distribution map. PV: peak viscosity; HPV: hot paste viscosity; CPV: cold paste viscosity; BD: breakdown; SB: setback; HD: hardness.

Rheological properties are core indices reflecting starch pasting stability and textual properties and directly affect the CEQ of rice. Peak viscosity (PV) reflects the maximum viscosity of starch granules during complete gelatinization and is an important index for evaluating the viscosity of rice during cooking. PV was mainly concentrated in the range of 225–325 RVU (Figure 3), with a minimum of 110.5 RVU, a maximum of 340.1 RVU and a mean of 267.5 RVU (Table 1). Hot paste viscosity (HPV) and breakdown value (BD) reflect the thermal stability of starch paste: the lower the HPV and the higher the BD, the poorer the thermal stability of starch paste but the softer and stickier the texture of cooked rice, and vice versa. HPV ranged from 34.1 to 224.8 RVU with a mean of 156.3 RVU, and BD ranged from 46.0 to 162.9 RVU with a mean of 111.2 RVU, indicating that most varieties had balanced thermal stability with certain variations among accessions. Setback value (SB) reflects the retrogradation characteristics of starch paste during cooling: the lower the SB, the slower the starch retrogradation rate, resulting in better taste of cold cooked rice with less hardness. SB ranged from −114.3 to 51.0 RVU with a mean of −32.3 RVU, showing extensive variation in starch retrogradation characteristics among the tested varieties (Table 1). Cool paste viscosity (CPV) reflects the viscosity of starch paste when cooled to 50 °C and is related to the cold viscosity and elasticity of cooked rice. CPV ranged from 55.9 to 341.1 RVU with a mean of 235.2 RVU, demonstrating that most rice varieties had moderate cold viscosity and balanced elasticity after cooking. Hardness (HD) is one of the important indices for evaluating the texture of cooked rice. The gel HD of the tested rice varieties ranged from 1.7 to 16.2 g with a mean of 6.3 g, mainly concentrated in the range of 2–8 g. The overall low-to-medium hardness level usually corresponds to a softer texture of cooked rice.

### 3.4. Gelatinization Properties

**Figure 4:**
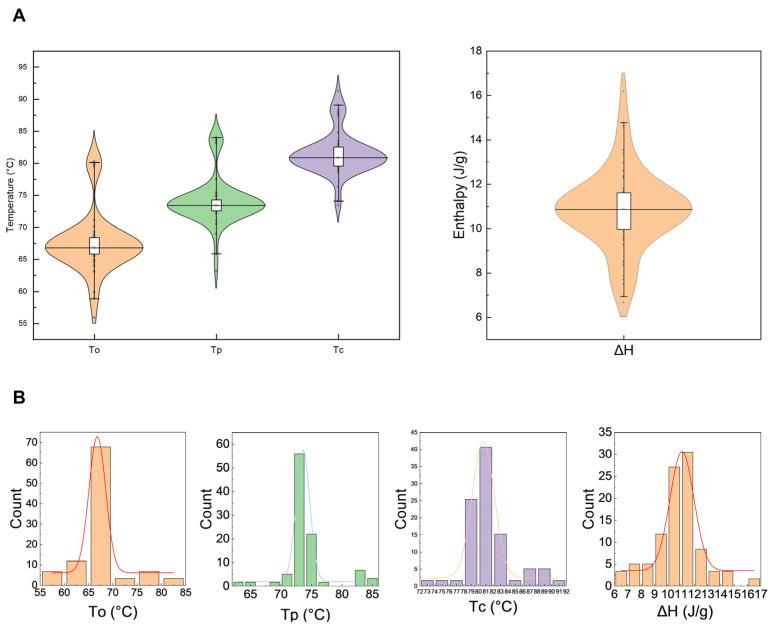
Distribution of the thermal properties, To, Tc, Tp, and ΔH, in different rice varieties. (**A**) Violin distribution map; (**B**) Histogram distribution map. To: onset temperature; Tp: peak temperature; Tc: conclusion temperature; ΔH: enthalpy change.

Starch gelatinization properties are core indices reflecting the stability of starch crystalline structure and the gelatinization process. The onset gelatinization temperature (To) was mainly concentrated in the range of 65–70 °C (Figure 4), with a range of 55.9–80.3 °C and a mean of 67.6 °C. The peak gelatinization temperature (Tp) was mostly concentrated in the range of 70–75 °C, with a range of 63.2–84.1 °C and a mean of 74.1 °C. The conclusion gelatinization temperature (Tc) was mainly concentrated in the range of 78–84 °C, with a range of 73.4–91.2 °C and a mean of 81.5 °C. In contrast to gelatinization temperature, the distribution of gelatinization enthalpy (ΔH) was more discrete (Figure 4). ΔH ranged from 6.6 to 16.2 J/g with a mean of 10.8 J/g (Table 1).

### 3.5. Correlation Analysis

**Figure 5:**
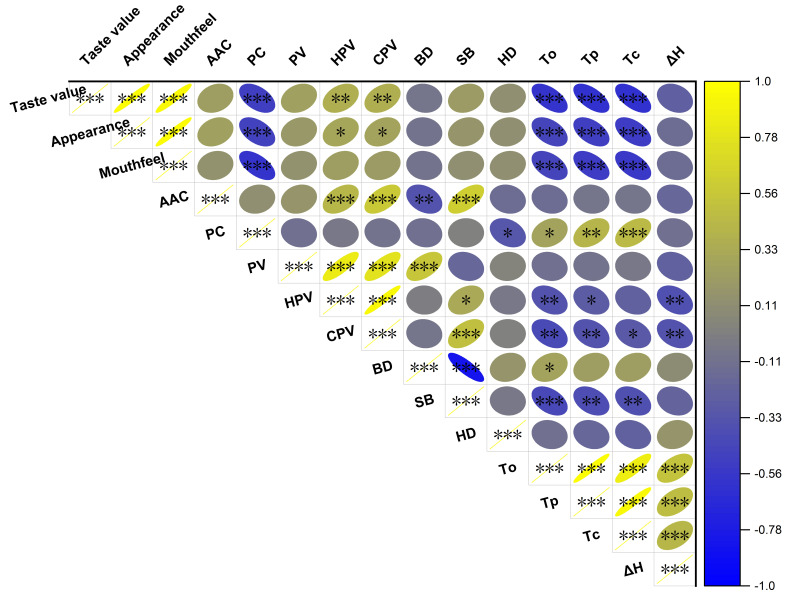
Correlation analysis of CEQ traits in different rice varieties. PV: peak viscosity; HPV: hot paste viscosity; CPV: cold paste viscosity; BD: breakdown; SB: setback; AAC: apparent amylose content; PC: protein content; HD: hardness; To: onset temperature; Tp: peak temperature; Tc: conclusion temperature; ΔH: enthalpy change. The heatmap displays Pearson correlation coefficients between CEQ traits, with color gradients indicating correlation strength (yellow for positive correlation, blue for negative correlation). Color intensity and circle size are proportional to the absolute value of the correlation coefficient. *, ** and *** indicate significant correlations at *p* ≤ 0.05, *p* ≤ 0.01 and *p* ≤ 0.001, respectively.

Pearson correlation analysis was performed on 15 quality traits, and the results showed that the taste value was negatively correlated with PC, To, Tc and Tp, but significantly positively correlated with appearance score, mouthfeel score, HPV and CPV (Figure 5). PC was positively correlated with To, Tc and Tp, and negatively correlated with HD. AAC was positively correlated with HPV, CPV and SB, and negatively correlated with BD. ΔH was positively correlated with To, Tp and Tc, and negatively correlated with HPV and CPV. Significant positive correlations were observed among the pasting parameters PV, HPV and CPV. BD was negatively correlated with SB.

### 3.6. Cluster Analysis

**Figure 6:**
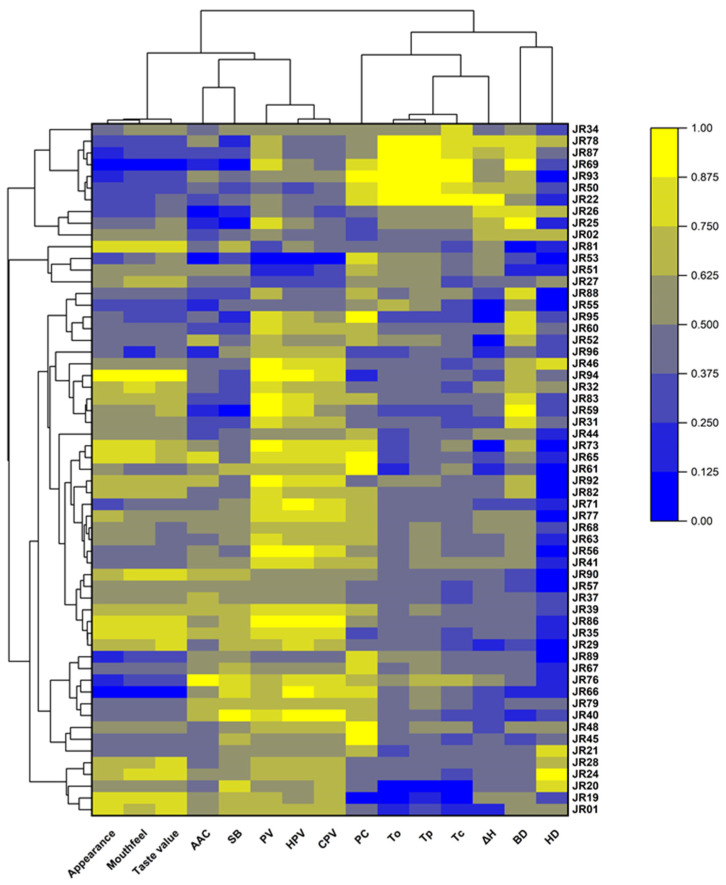
Heatmap of cluster analysis for quality traits in different rice varieties. PV: peak viscosity; HPV: hot paste viscosity; CPV: cold paste viscosity; BD: breakdown; SB: setback; AAC: apparent amylose content; PC: protein content; HD: hardness; To: onset temperature; Tp: peak temperature; Tc: conclusion temperature; ΔH: enthalpy change. Color gradients represent the relative content or level of each CEQ trait (yellow indicates high levels; blue indicates low levels), with color intensity visually reflecting the relative differences among CEQ traits. Each row represents a different rice variety, and each column represents a different CEQ trait.

Cluster analysis revealed the differences and similarities in various quality traits among the 59 rice varieties. Based on the characteristics of cooking and eating quality (CEQ) traits, the tested rice varieties were divided into two clusters (Figure 6). Cluster 1 included 10 varieties, which exhibited higher gelatinization temperature parameters (To, Tc, and Tp), gelatinization enthalpy (ΔH), PC and BD, as well as lower taste value, AAC, PV, HPV, CPV and SB. The remaining 49 rice accessions were grouped into Cluster 2, which showed the opposite characteristics to those of Cluster 1: higher taste value, AAC, PV, HPV, CPV and SB, and lower To, Tc, Tp, ΔH and BD. Clearly, this cluster could be further divided into multiple sub-groups. Cluster analysis at the trait level showed that appearance score, mouthfeel score, taste value, AAC, SB, PV, HPV and CPV were clustered into one group, while PC, To, Tc, Tp, BD and HD were clustered into another group, which was consistent with the results of correlation analysis.

### 3.7. Principal Component Analysis (PCA)

**Figure 7:**
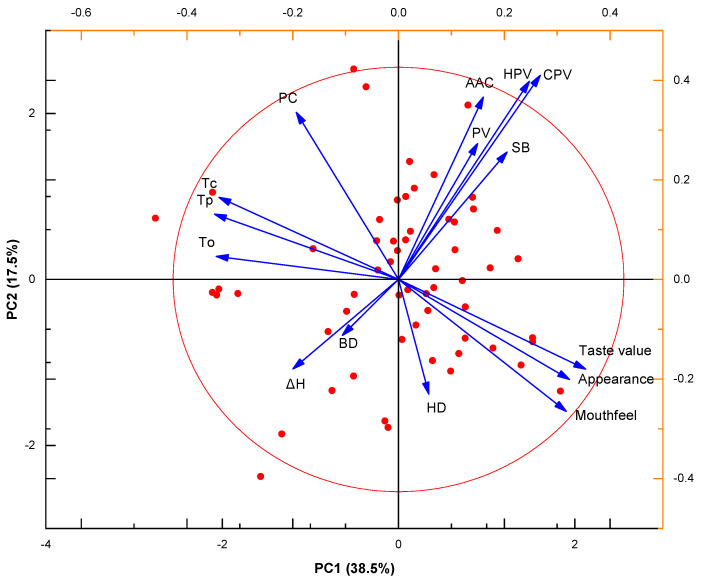
Principal component analysis of CEQ traits in different rice varieties. PV: peak viscosity; HPV: hot paste viscosity; CPV: cold paste viscosity; BD: breakdown; SB: setback; AAC: apparent amylose content; PC: protein content; HD: hardness; To: onset temperature; Tp: peak temperature; Tc: conclusion temperature; ΔH: enthalpy. Red scatter points represent samples from different rice varieties. Blue arrows indicate the direction and length of contribution from different CEQ traits to the principal component, respectively. Ellipses denote 95% confidence intervals.

PCA simplifies the complex correlations among multiple traits through dimensionality reduction and can intuitively present the relationship between different rice varieties and CEQ traits. As shown in the PCA plot (Figure 7), the 95% confidence ellipse covered the vast majority of samples. The first principal component (PC1) explained 38.5% of the total variation, and the second principal component (PC2) explained 17.5%, with a cumulative explanation of 56.0% of the total variation by PC1 and PC2. This indicated that PC1 and PC2 could effectively summarize the core variation information of 15 CEQ traits. PC1 was mainly driven by AAC, HPV, CPV, PV, SB, taste value, appearance score, and mouthfeel score, which showed strong positive loadings. In contrast, PC and gelatinization temperatures (To, Tp, and Tc) exhibited strong negative loadings on PC1, representing the most influential traits responsible for sample separation. The vector for taste value pointed in the opposite direction to those of PC, To, Tp and Tc, visually confirming that low PC and low gelatinization temperatures were strongly associated with high eating quality. This distinct directional separation in the PCA plot clearly differentiated rice varieties: accessions with higher taste value, higher AAC, higher paste viscosities (HPV and CPV), lower PC and lower gelatinization temperatures clustered on one side, while varieties with the opposite trait profiles clustered on the other side. 

### 3.8. Multiple Linear Regression Analysis and Path Analysis

To more accurately predict the taste value with the physicochemical properties, stepwise regression analysis with significance testing was employed to construct the optimal fitting equation for taste value. The best regression equation was Taste value = 142.526 − 5.226 PC − 0.425 To (R^2^ = 0.455; *p* < 0.001) (Table 2). The analysis identified total protein content (PC) and onset temperature of gelatinization (To) as the core parameters affecting taste values, explaining 45.5% of the total variation. Therefore, path analysis was further conducted to quantify the direct and indirect contributions of PC and To to taste values by decomposing correlation coefficients, thereby identifying core determinants and their regulation pathways (Table 3). PC exhibited the strongest direct negative effect on taste value (direct path coefficient = −0.5090), with a very small indirect effect via To (indirect path coefficient = −0.0887). Meanwhile, To also negatively affected taste value through its direct effect (−0.3251) and indirect effect via PC (−0.1389). This is consistent with the significant negative correlation between taste value and PC and To observed in correlation analysis, indicating that both PC and To play critical roles in determining rice taste value through direct and indirect pathways.

## 4. Discussion

### 4.1. Chemical Basis of Eating Quality of Rice

Chemical components are the intrinsic core factors determining rice eating quality. Starch is the primary storage substance in rice endosperm, accounting for 72% to 82% (dry basis) of brown rice and 90% of milled rice [4]. Starch consists of amylose and amylopectin. Apparent amylose content (AAC) is the most important factor affecting rice eating quality: the higher the AAC, the lower the taste value and the poorer the rice eating quality [24,25]. The fine structure of amylopectin is another crucial factor influencing rice eating quality [13,15]. Studies have shown that rice with a higher proportion of amylopectin short chains and a lower proportion of long chains had better cooking and eating quality [15,17]. For hybrid rice varieties with similar AAC, a higher proportion of amylopectin short chains and a lower proportion of long chains result in higher peak viscosity, breakdown value, and a soft and sticky texture of cooked rice [17]. Starch with a higher gelatinization temperature has a more stable crystalline structure, resulting in relatively harder cooked rice [14]. Waxy rice varieties have a lower distribution of amylopectin medium and long chains and a higher distribution of short chains, leading to lower RVA profile characteristic values and GT [25]. In this study, the mean AAC of the tested varieties was 15.5%, and 93.2% of the varieties had low AAC in the range of 10–20% (Table 1; Figure 2), which is the optimal AAC range for high-quality japonica rice [14]. Correlation analysis showed that AAC was significantly positively correlated with setback value (SB) and significantly negatively correlated with breakdown value (BD) (Figure 5). This was consistent with most previous results using rice with a wide diversity in AAC [18,25,26,27,28]. The effects of AAC on eating quality and textural properties are well documented, with low amylose levels usually associated with tender, cohesive, glossy cooked rice, while higher amylose cultivars tend to cook dry, be fluffy and separate [29]. In this study, however, the small variation of AAC means that it has no correlation with the taste value (Figure 5).

There is a significant negative correlation between PC and rice eating quality, with rice varieties with lower PC showing higher taste values [7]. Most studies have shown that PC is significantly negatively correlated with gel consistency, breakdown viscosity, peak viscosity and taste value, and significantly positively correlated with setback value [7]. However, when PC ranges from 6.61% to 9.34%, it alone is insufficient to reflect rice eating quality accurately [8]. The varieties with good taste value had higher albumin and lower globulin and glutelin content [15]. In this study, the mean total PC of rice in this study was 8.0%, which fell within the range of 6.61–9.34% (Table 1; Figure 2). A significant negative correlation was observed between PC and taste value, which was in agreement with previous reports [3,8]. However, PC may indirectly regulate eating quality through its impact on starch gelatinization temperature and grain structure [3]. The protein binds closely with starch granules in rice grain with high PC; this impact structure results in an increase in the gelatinization of starch and a decrease in the degree of doneness of cooked rice [3]. The glutelin content, a major component of rice protein, also had a highly significant negative correlation with taste value, which may affect the hardness of cooked rice [3]. The globulin content also has an impact on the rice CEQ [15,30,31]. However, Matsue et al. [21] reported that protein and amylose contents had no correlation with eating quality. Because their studies all employed japonica rice, the environmental effect on protein and amylose content cannot be excluded. The temperature during rice growth and application of fertilizers, particularly nitrogen, have significant effects on the PC and eating quality of rice [31,32,33]. Xu et al. [11] demonstrated that growing location strongly influenced the eating quality of japonica rice, with overall eating quality negatively correlated with PC and positively correlated with gel hardness, supporting the key roles of protein- and texture-related properties in eating quality evaluation.

### 4.2. Correlation Analysis Between Starch Physicochemical Properties and Taste Value

Starch pasting properties and gelatinization properties reflect the starch gelatinization behavior from the perspectives of macroscopic rheology and microscopic crystalline structure, respectively, and there is a close intrinsic relationship between them. The results of this study showed that HPV and CPV were significantly positively correlated with the taste value, which was consistent with the results of Bao et al. [2]. Peng et al. [17] indicated that a higher PV and BD is associated with a softer and stickier texture of cooked rice, but there was no significant correlation between BD and the taste value in this study. This could be attributed to the inherent differences in genetic background and starch structure between conventional japonica varieties and hybrid rice lines. Hybrid rice has a wide range of variation in AAC and amylopectin fine structure, which leads to a large range of variation in BD and makes BD a sensitive indicator for distinguishing eating quality. In contrast, the 59 japonica rice varieties used in this study had a narrow and concentrated AAC range (10–20%), with possibly homogeneous amylopectin chain length distribution and crystalline structure, thus resulting in a small variation in thermal stability (BD) and weakened correlation with taste value. The mean SB of the tested japonica rice was −32.3 RVU, and most varieties exhibited low starch retrogradation characteristics, which was consistent with the results of Wang et al. [25]. A low SB value indicates that cold cooked rice is less likely to become firm during cold storage and thus tastes better. This characteristic is an important factor that makes the eating quality of japonica rice superior to that of other rice types.

Starch thermodynamic properties reflect the stability of the starch crystalline structure, and their correlation with the taste value directly reflects the effect of crystalline structure on rice cooking and eating quality. In this study, the To, Tp and Tc were significantly negatively correlated with the taste value, indicating that japonica rice varieties with a lower gelatinization temperature had better eating quality. This is because starch with a lower gelatinization temperature has a looser crystalline structure, which is more easily hydrated and gelatinized during cooking, resulting in a softer texture of cooked rice [14]. ΔH is significantly positively correlated with gelatinization temperature, and its value reflects the crystallinity of starch: a smaller ΔH indicates lower starch crystallinity and less energy required for gelatinization, resulting in better palatability of cooked rice [27].

Texture properties are closely linked to both starch components and mouthfeel. Zhao et al. [34] reported that cooked soft japonica rice had significantly lower hardness due to low AAC, which aligned with the negative correlation between gelatinization temperature and taste value in the present study. Meanwhile, the instrumental texture parameters (e.g., hardness) obtained from rice gel were highly consistent with texture attributes, verifying the reliability of physicochemical indicators in predicting eating quality [11].

In addition, besides the physicochemical properties mentioned above, flavor is also an important characteristic of rice since it is a preferred trait by consumers [35]. Zhao et al. [34] identified thirteen and eighteen volatile markers distinguishing soft and non-soft rice in raw and cooked rice, respectively, with headspace solid-phase micro-extraction gas chromatography–mass spectrometry (HS-SPME-GC-MS). However, in this study, we did not conduct experiments related to aromatic compounds, which is a limitation, and we will conduct supplementary experiments in a future study.

### 4.3. Application of Multivariate Analysis in the Evaluation of Rice Eating Quality

Due to the large number of rice quality traits and their complex interrelationships, evaluation based on a single index is often limited. Correlation analysis in this study found that taste value was significantly negatively correlated with total PC and gelatinization temperature parameters and significantly positively correlated with appearance score, mouthfeel score, and pasting parameters (HPV and CPV). Although these correlations reveal the pairwise relationships between traits, they do not fully capture the combined effects of multiple traits. Principal component analysis (PCA) reduces multiple complex traits to a few principal components. In this study, PC1 and PC2 cumulatively explained 56.0% of the total variation. The analysis results showed that AAC, HPV, CPV, PV and SB were the key traits contributing to PC1 variation, and the arrow direction of the taste value was completely opposite to that of total PC, To, Tc and Tp. Chen et al. [18] also indicated that the RVA profile was affected by protein and amylose contents and explained 60.5% of the taste value variation in indica rice. Multiple regression analysis indicated that PC and To have direct and indirect effects on taste value (Table 2). Because japonica rice has narrow variation in AAC while PC shows wider variation, PC thus plays a more important role than AAC. Even in indica rice, PC explained 38.6% of the variation in taste value [18]. The prediction formula derived from PC and To can be used to quickly predict the taste value based on physicochemical properties. However, for accurate evaluation of eating quality, both instrument-based taste value and sensory-panel-based quality should be used to verify the eating quality.

However, the R^2^ = 0.455 from the multiple linear regression analysis indicates that more than 50% of the variation in taste scores remains unexplained. It is speculated that this is due to the presence of variables not included in the analytical model but which may have a critical impact on taste scores. For example, the fine structure of branched-chain starch [13,15,17], lipid content and fatty acid composition [3,36], volatile markers [34], the proportion of protein components [15,30,31] and the morphology and surface characteristics of starch granules [14,17] are also key factors influencing the taste quality of rice. Future studies could determine starch chain length distribution, lipid content, fatty acid composition, and the levels of albumin, globulin, glutenin, and alcohol-soluble proteins. Incorporating these parameters into multiple linear regression analysis would further refine the equation determining the taste score of japonica rice.

### 4.4. Limitations of the Study

This study provides a systematic physicochemical basis for understanding the eating quality of japonica rice, yet several limitations should be acknowledged for further investigation. Firstly, all rice materials were planted in only one experimental site and harvested in a single cropping season. The effects of genotype × environment (G × E) interaction [32], climatic variation (high temperature) [37], soil conditions and nitrogen fertilizer [38,39,40,41,42] on eating-quality-related traits were not evaluated, which may restrict the universality of the present conclusions. Secondly, eating quality was solely determined using a rice taste analyzer, without verification by a trained human sensory panel. Although instrumental taste value is highly correlated with sensory traits, direct human panel data (e.g., stickiness, elasticity, aroma, and overall palatability) would strengthen the reliability and practical relevance of the results. In addition, the AAC of the 59 japonica varieties ranged narrowly from 9.4% to 22.1%, with most samples concentrated in 10–20%. The limited variation in AAC restricted the ability to evaluate its full impact on eating quality, especially for ultra-low- or ultra-high-AAC rice types. Lastly, the regression model established for taste value prediction was based only on the modeling dataset from this study, without validation using an independent set of rice varieties. Thus, the stability and accuracy of the predictive equation across different genetic backgrounds remain to be verified.

## 5. Conclusions

In this study, taste value, protein content, amylose content, pasting properties, gelatinization properties, and textural properties were systematically determined in 59 japonica rice varieties, with the aim of identifying physicochemical properties that can predict taste value. Wide genotypic variation was observed among the traits analyzed. Multiple regression analysis indicated that protein content and onset gelatinization temperature served as core parameters for predicting the taste value of japonica rice, explaining 45.5% of the variation in taste value. This study provides a rapid prediction of japonica rice quality based on physicochemical properties and lays a solid foundation for the genetic improvement and breeding of high-quality japonica rice varieties.

## Figures and Tables

**Table 1:** Summary of cooking and eating quality traits for different rice varieties.

Traits	Mean ± SD	Min	Max	CV (%)	Skewness	Kurtosis
Appearance score	6.1 ± 1.0	3.5	8.5	16.42	−0.14	−0.17
Mouthfeel score	6.7 ± 0.9	4.3	8.8	13.35	−0.14	−0.03
Taste value	72.0 ± 6.4	54.0	87.8	8.94	−0.16	0.47
PC (%)	8.0 ± 0.6	6.1	9.2	7.83	−0.38	0.24
AAC (%)	15.5 ± 2.3	9.4	22.1	14.79	−0.30	1.26
To (°C)	67.6 ± 4.9	55.9	80.3	7.27	1.15	2.42
Tp (°C)	74.1 ± 3.8	63.2	84.1	5.17	0.96	2.98
Tc (°C)	81.5 ± 3.3	73.4	91.2	4.04	0.81	1.83
ΔH (J/g)	10.8 ± 1.8	6.6	16.2	16.60	0.19	1.28
PV (RVU)	267.5 ± 43.6	110.5	340.1	16.29	−1.14	2.30
HPV (RVU)	156.3 ± 36.7	34.1	224.8	23.51	−0.61	0.89
BD (RVU)	111.2 ± 24.2	46.0	162.9	21.79	−0.16	−0.05
CPV (RVU)	235.2 ± 49.4	55.9	341.1	21.00	−0.88	1.88
SB (RVU)	−32.3 ± 32.7	−114.3	51.0	−101.27	−0.22	0.23
HD (g)	6.3 ± 3.4	1.7	16.2	53.70	1.03	0.39

CV: coefficient of variation; PV: peak viscosity; HPV: hot paste viscosity; CPV: cold paste viscosity; BD: breakdown; SB: setback; AAC: apparent amylose content; PC: protein content; HD: hardness; To: onset temperature; Tp: peak temperature; Tc: conclusion temperature; ΔH: enthalpy.

**Table 2:** The multiple linear regression equation between taste value and physicochemical parameters of starch.

Trait	Multiple Linear Regression Equation	*R* ^2^	Adjusted *R*^2^	*F*	*P*	VIF_PC_	VIF_To_
Taste value	Taste value = 142.526 − 5.226 PC − 0.425 To	0.455	0.436	23.378	<0.001	1.080	1.080

VIF: variance inflation factor.

**Table 3:** Path analysis of the effects of starch physicochemical parameters on taste value.

Trait	Factor	Simple Correlation Coefficient	Direct Path Coefficient	Indirect Path Coefficient			Decision Coefficient
				PC	To	Total	
Taste value	PC	−0.5977	−0.5090		−0.0887	−0.0887	0.3494
	To	−0.4639	−0.3251	−0.1389		−0.1389	0.1959

## Data Availability

The original contributions presented in this study are included in the article/Supplementary Materials. Further inquiries can be directed to the corresponding authors.

## Supplementary Materials

The following supporting information can be downloaded at https://www.mdpi.com/article/10.3390/foods15101689/s1. Table S1. The japonica rice accessions used in this study.
