# Supplementary material for: Evaluation of Eating Quality in Japonica Rice: A Multi-Trait Analysis of Starch Properties, Protein Content and Taste Value
Source: Foods. 2026 May 12;15(10):1689. doi: 10.3390/foods15101689 (PMC13205976; doi:10.3390/foods15101689)
Supplement: Supplementary file 1 [file foods-15-01689-s001.zip › foods-4278721-supplementary.pdf]

Table S1. The japonica rice accessions used in this study

Code	Variety name
JR01	Zhedazaojingdao
JR02	Youhao2
JR19	Honghuakemi
JR20	Baimaogeng
JR21	Gangyou 1
JR22	Yanfeng
JR24	Jinongda 168
JR25	Nanjing 9108
JR26	Jinjing 818
JR27	Xiaolixiang
JR28	Songjing 22
JR29	Jijing 830
JR31	Yanfeng 47
JR32	Shennong 625
JR34	Wuyoudao 4
JR35	Suijing 15
JR37	Suijing 17
JR39	Longjing 31
JR40	Zhongkefa 5
JR41	Jinhe 1
JR44	Chaojidao
JR45	Suijing 27
JR46	TL 619
JR48	Panyu rice
JR50	Liaojing 401
JR51	Huaidao 5
JR52	Gangyu 2
JR53	Nanjing 5718
JR55	Jinongda 505
JR56	Liaojing 1402
JR57	Qijing 2
JR59	Nanjing 46
JR60	Jinhe 2
JR61	Liaoxing 1
JR63	Longjing 40
JR65	Tianlongjing 311
JR66	Lianjing 16
JR67	Liaoxing 8
JR68	Tiejing 17
JR69	Dongyandao 18
JR71	Yanjing 48
JR73	Fujing 289
JR76	Nanjing 3908
JR77	Longjing 65
JR78	Gangyuan 97
JR79	Meifengdao 331

JR81	Hongke 389
JR82	Longjing 29
JR83	Panjing 968
JR86	Lianhui 6730
JR87	Gangyou 3
JR88	Kenyan 017
JR89	Shenxingdao 2
JR90	Suijing 22
JR92	Weiming-1
JR93	Weiming-2
JR94	Koshihikari
JR95	Zhejing 7B
JR96	ZJ157
